# A Portion of the Apomixis Locus of *Paspalum Simplex* is Microsyntenic with an Unstable Chromosome Segment Highly Conserved Among Poaceae

**DOI:** 10.1038/s41598-019-39649-6

**Published:** 2019-03-01

**Authors:** Giulio Galla, Lorena A. Siena, Juan Pablo A. Ortiz, Helmut Baumlein, Gianni Barcaccia, Silvina C. Pessino, Michele Bellucci, Fulvio Pupilli

**Affiliations:** 1 Department of Agriculture Food Natural resources Animals and Environment (DAFNAE), University of Padova, 35020 Legnaro (PD), Italy; 2 Instituto de Investigaciones en Ciencias Agrarias de Rosario (IICAR), CONICET-UNR, Laboratorio de Biología Molecular, Facultad de Ciencias Agrarias, Universidad Nacional de Rosario, S2125ZAA Zavalla, Argentina; 3 The Leibniz Institute of Plant Genetics and Crop Plant Research (IPK), D-06466 Gatersleben, Germany; 4 Institute of Biosciences and Bioresources (IBBR), National Research Council (CNR), 06128 Perugia, Italy

## Abstract

The introgression of apomixis in major seed crops, would guarantee self-seeding of superior heterotic seeds over generations. In the grass species *Paspalum simplex*, apomixis is controlled by a single locus in which recombination is blocked. In the perspective of isolating the genetic determinants of apomixis, we report data on sequencing, *in silico* mapping and expression analysis of some of the genes contained in two cloned genomic regions of the apomixis locus of *P*. *simplex*. *In silico* mapping allowed us to identify a conserved synteny group homoeologous to the apomixis locus, located on a telomeric position of chromosomes 12, 8, 3 and 4 of rice, *Sorghum bicolor*, *Setaria italica* and *Brachypodium distachyum*, respectively, and on a more centromeric position of maize chromosome 1. Selected genes of the apomixis locus expressed sense and antisense transcripts in reproductively committed cells of sexual and apomictic ovules. Some of the genes considered here expressed apomixis-specific allelic variants which showed partial non-overlapping expression patterns with alleles shared by sexual and apomictic reproductive phenotypes. Our findings open new routes for the isolation of the genetic determinants of apomixis and, in perspective, for its introgression in crop grasses.

## Introduction

The Poaceae angiosperm family, commonly referred to as grasses, accounts for about 70% of crops in the world. Although frequent events of genomic re-patterning and gene losses, especially occurring after whole genome duplication (WGD) events, caused considerable divergence in genome size and chromosome number^1,2^, genes tend to maintain their position in the same chromosome region with strict conservation of gene order (collinearity) or without it (synteny)^3^. Genomic collinearity between grasses is correlated with conservation of expression between orthologous gene pairs^4^. Therefore, comparative analysis of conservation of gene position makes it possible: (*i*) to get insights into the evolution of specific genes or entire metabolic networks and signalling pathways^5^; (*ii*) to identify niche specific genes important for adaptation^6^; (*iii*) to unveil large scale genomic events such as WGDs^7^; and, (*iv*) to link phenotypic traits with genotypic properties, thereby improving gene discovery^8^.

The genus *Paspalum*^9^ Panicoideae is characterized by an extraordinary plasticity of the reproductive system^10^, including strict outcrossing, a variable extent of allowed self-compatibility, and nearly obligate apomixis, *i*.*e*. asexual reproduction by seeds^11^. Introgression of apomixis to crops would allow the fixation of heterosis and therefore the re-seeding of hybrid F1 seeds without vigour loss^12^. The three main biological components of apomixis in *Paspalum* are: (1) apospory (*i*.*e*. unreduced embryo sac development from nucellar cells of the ovule without meiosis); (2) parthenogenesis (*i*.*e*. fertilization-free embryo development); and (3) unbalanced endosperm formation (*i*.*e*. capacity to form endosperm deviating from the canonical 2 maternal: 1 paternal genome ratio, allowing a maternal contribution excess). These components are controlled by a single complex dominant superlocus (Apomixis Controlling Region or ACR)^10^. Comparative genetic mapping efforts in *Paspalum* evidenced various extents of synteny of the ACR with the subtelomeric part of rice chromosome 12 long arm^13–16^. The ACR of *P*. *simplex* revealed structural features of heterochromatin, namely presence of transposable elements (TEs), gene degeneration^17^ and deregulation^18^. One ACR-specific pseudogene, *PsORC3*, constitutively expressed a non-coding RNA that silenced its functional counterpart via a sense-antisense mechanism^19^. As the rice homolog of this gene did not map on the distal end of chromosome 12, it was assumed that it migrated to the ACR from other locations after rice and *Paspalum* diverged from a common ancestor.

Although rice is considered the best-suited reference genome for comparative genomics in grasses^20^, this species is phylogenetically rather distant from *Paspalum*^21^. Nowadays, genome sequences representative of the most economically important grass clades, such as Panicoideae including *Sorghum*, maize and *Setaria* and Poideae with *Brachypodium* are publicly available^22–25^. Among these, the species of particular interest for comparisons with *Paspalum* are *Sorghum bicolor* (L.) and *Setaria italica* (L.) P. Beauv., as both of them diverged from the *Paspalum* clade ca. 10 mya^26^, thus representing the two most closely related genomes for which Whole Genome Sequencing (WGS) information is available. Although the apospory-specific genomic region (ASGR) of other grasses, such as *Pennisetum squamulatum* syn. *Cenchrus ciliaris*, shares many structural similarities with the *Paspalum* ACR, *i*.*e*. repression of recombination, accumulation of repetitive elements and gene degeneration, no relevant large-scale collinearity and/or synteny between the ASGR and reference genomes (rice, *Setaria* and *Sorghum*) were detected so far^27^. Although comparative mapping of apomixis within the *Paspalum* genus revealed a low extent of gene movement and divergence at the ACR even among very closely related species^15,16^, a portion of the same region, identified by markers of rice chromosome 12 was linked to apomixis in all apomictic species of *Paspalum* analysed to date^15^. We argued that markers not linked to the ACR in all species of *Paspalum* belong to genomic regions that are dispensable for the expression of the trait, whereas all those linked to apomixis in multiple *Paspalum* spp. delineate a genomic portion in which the essential genetic determinants of the trait are likely located. Bearing this in mind, we investigated on whether larger areas of synteny could be identified between the ACR of *P*. *simplex* and specific regions of grasses (*i*.*e*. *Sorghum*, maize, *Brachypodium* and *Setaria*) that are more closely related to *Paspalum* compared to rice.

The aim of this research was to study the genomic arrangement of ACR portions in *P*. *simplex* at both structural and functional levels. The specific aspects we wanted to address were: *i*) to disclose more extensive areas of homology between the ACR of *P*. *simplex* and the closely related genomes of *S*. *italica*, *S*. *bicolor* and maize together with those of the more distantly related *Brachypodium* and rice as controls; and *ii*) to verify the existence of relationships between degeneration of the the apomixis-linked genes, and possible silencing effect on their homologues.

## Results

### Comparative mapping

**Figure 1:**
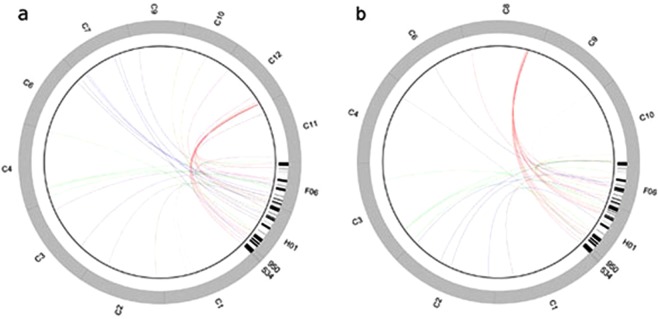
Mapping of genes contained in apomixis-linked BACs on chromosomes of (**a**) *Oryza sativa* and (**b**) *Sorghum bicolor*. F06, H01, 950 and 534 correspond to contigs PS127F6_c1, PS366H1_c1, H10_950 and H10_534, respectively. C1-C12 represent the chromosome number for each genome. Genes providing alignments with e-value < 1.0e^−9^ were plotted as black bars. Otherwise, gene regions were plotted as grey bars. Red lines link genes to the conserved chromosome area related to apomixis revealed in this study.

**Figure 2:**
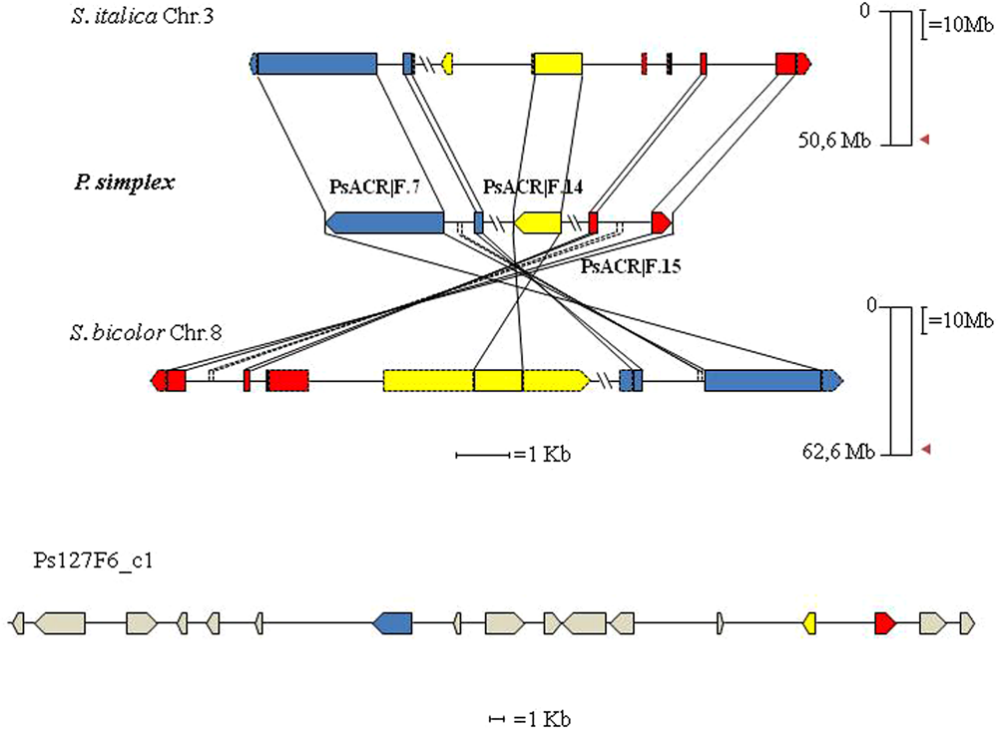
Diagram of microcollinearity of the genes contained in the contig PS127F6_c1 with the conserved area of homology in *Setaria italica* and *Sorghum bicolor* genomes. Position of the apomixis-linked chromosome areas are highlighted on the right. Vertical continuous and dotted lines connect homologous coding and non-coding regions respectively, full boxes identify exons, dotted boxes UTRs, and horizontal lines refer to introns and/or intergenic regions. Each gene is represented by one colour with its orientation indicated by the arrow head. Ps127F6_c1 identify the *P*. *simplex* whole contig; coloured boxes identify genes mapped to the apomixis related area of *Sorghum* and *Setaria* whereas those coloured in grey mapped elsewhere in the same genomes.

To identify BAC clones containing the genetic determinants of apomixis, 39 out of the 41 BACs positive to the SCAR markers co-segregating with apomixis^28^ were hybridised with marker-derived probes delineating the sub-portion of the ACR linked to apomixis in several *Paspalum* species (c1069, c454 and c996)^15^. Of these, only c996 showed a clear signal in two overlapping clones (127F6 and 296A7; Supplementary Fig. S1), as these were selected with the same SCAR marker^28^. The other two BACs 333G1 and 312H12, included in the same contig^28^_,_ showed a less intense signal likely due to either background signal (see below) or partial hybridisation with the labelled probe. Background signals in spots not related to 127F6, 296A7, 333G1 and 312H12 are due to unspecific hybridisation of template BAC vector DNA with residual labelled plasmid DNA vector still present in the probe mixture. Then, among these BACs only 127F6 was chosen for sequencing. The other BAC considered in this study (366H1) was isolated previously with the AFLP-derived SCAR marker EM 180^29^, which mapped in the same sub-portion of the ACR^15^. Both BAC clones were sequenced at 6× coverage. Finally, 6 and 5 contigs whose length ranged from 976 to 73,550 bp and from 9,403 to 75,967 bp were assembled for 127F6 and 366H1, respectively. Various transposon elements (TE)-related sequences (Supplementary Table S1) were identified in the considered contigs covering from 12% (PS127F6_c1) to 20.40% (PsH10) (Table 1) of the total length analysed. No relevant differences were detected between apomixis-linked contigs reported in this study and those previously analysed (PsH10)^17^ nor with the contig (Ps366H1_c5) containing the hemizyigous SCAR marker used to isolate the related BAC. Retrotransposons of the LTR gypsy and Copia subclasses were the most abundant, whereas among transponsons, elements related to Helitron subclasses were the most frequent. Proportions of simple and low complexity repeats were highly similar in all 4 query sequences. Most of the apomixis-linked genes annotated on TAIR database are depicted to DNA/RNA binding molecular process (Supplementary Table S1). The largest contig assembled for each BAC was analyzed in detail for gene synteny and collinearity with five reference grass genomes. The contig PS127F6_c1 of the BAC 127F6 contained 50 ORFs organized as 15 predicted genes and 2 pseudogenes (the latter so defined by the presence of one or more premature stop codons on their CDS) named PsACR|F.1–17 (Supplementary Table S2). However only 8 of them showed significant homology (e value ≤ 1.0e[−9]) with annotated genes in GRAMENE database and, of these, only 4 in TAIR repository. The gene PsACR|F.15 was homologous to the c996 EST rice marker (OS12G0616200) used to select the BAC. The position of this gene in the rice map marks the telomeric end of the rice region homologous to the ACR common to several *Paspalum* spp.^15^. However, the gene PsACR|F.7 was homologous to a more telomeric rice gene (OS12G0616500), suggesting that the ACR of *Paspalum* might be larger than that estimated previously. Thus, in this contig a region spanning 39,587 bp bracketed by the two genes PsACR|F.7 and PsACR|F.15 was syntenic with a portion of similar size of the rice genome located on chromosome 12 (39,515 bp, Supplementary Table S2 and Fig. 1a). However, as the rice gene OSG12G0616400, located between OSG12G0616200 and OSG12G0616500 (http://www.gramene.org/), has been replaced in *Paspalum* by seven genes whose rice homologs did not belong to a specific syntenic block (PsACR|F.8–14; Supplementary Table S2; Fig. 2), gene collinearity between rice and ACR has not been respected in this area. Looking at the other reference grass genomes we noticed that the ACR of *Paspalum* pointed specific areas of homology to a telomeric region of chromosomes 8 of *Sorghum* (Fig. 1b), 3 of *Setaria* and 4 of *Brachypodium*, and to a more centromeric region of chromosome 1 of maize (Supplementary Table S2). Similarly to rice, synteny but not collinearity was detected to the region of *Sorghum* delineated by the three genes SORBI_008G172500, SORBI_008G172200 and SORBI_008G172100 (homologous of genes PsACR|F.7, PsACR|F.14 and PsACR|F.15 respectively; Supplementary Table S2). Conversely, the homologous area of *Setaria* maintained all the homologs (SETIT_021254m.g., SETIT_022811m.g. and SETIT_023449m.g.) in perfect collinearity with the same genes of *P*. *simplex* (Supplementary Table S2; Fig. 2). Overall, homology inferred from statistically significant similarity was detected between predicted exons of *Paspalum* genes and their homologs in grass genomes, whereas no significant homology was detected for the intronic regions with the exception of last intron of the genes PsACR|F.7 and PsACR|F.15 and the related genes of *Sorghum* only (Fig. 2). Although the reciprocal orientation of genes was conserved between *Paspalum* and grasses, the whole region was inverted in *Sorghum*, with respect to *Paspalum* and *Setaria* (Fig. 2).

**Table 1:** Analysis of repetitive elements in apomixis-linked BACs of *P*. *simplex*.

Type of repeats	Repeat class/family	Query sequence (length)							
		Ps127F06_c1 (73,550)		Ps366H1_c1 (75,957)		Ps366H1_c5 (11,754)		PsH10 (75,005)	
Retroelements		N	Length occupied (%)	N	Length occupied (%)	N	Length occupied (%)	N	Length occupied (%)
	LINE/L1			1	39 (0.05)	2	1,471 (12.51)	2	1,621 (2.16)
	LTR/Gypsy	9	4,355 (5.92)	16	3,696 (4.87)			6	1,260 (1.68)
	LTR/Copia	1	69 (0.09)	4	5,381 (7.08)			8	8,923 (11.90)
**Transposons**									
	DNA/TcMar-Stowaway			2	418 (0.55)			2	433 (0.58)
	RC/Helitron	4	2,736 (3.72)	2	173 (0.23)			1	143 (0.19)
	DNA/MuLE-MuDR	1	11 (0.01)						
	DNA/PIF-Harbinger	2	554 (0.75)			1	98 (0.83)		
	DNA/hAT-Ac	2	381 (0.52)						
	DNA/hAT-Tip100							2	1,580 (2.11)
	DNA/PIF-Harbinger	2	554 (0.75)					1	932 (1.24)
	DNA/CMC-EnSpm	1	166 (0.23)					3	409 (0.55)
Total interspersed repeats			8,826 (12)		9,707 (12.8)		1,569 (13.35)		15,301 (20.40)
Simple repeats		25	1,308 (1.78)	19	852 (1.12)	4	242 (2.06)	12	851 (1.13)
Low complexity		1	39 (0.05)	5	214 (0.28)	1	60 (0.51)	1	47 (0.06)

**Figure 3:**
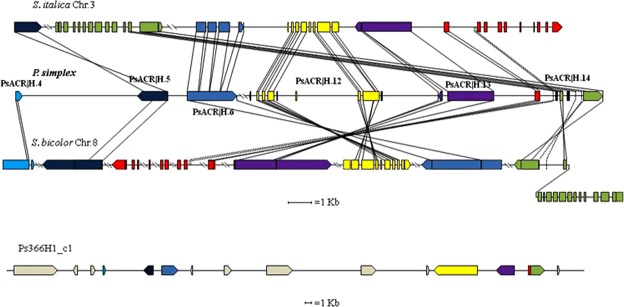
Diagram of microcollinearity of the genes contained in the contig PS366H1_c1 with the conserved area of homology in *Setaria italica* and *Sorghum bicolor* genomes. Symbols are as in Fig. 2.

The analysis of the largest contig of the BAC 366H1 (PS366H1_c1) highlighted the presence of 10 genes and 5 pseudogenes of which 8 and 4 showed highly significant homology to annotated genes in GRAMENE and TAIR repositories respectively (PsACR|H.1–15; Supplementary Table S2). Among the predicted genes, we found another group of genes/pseudogenes that pointed on the same chromosome regions that were located by the previous group of *Paspalum* genes (Supplementary Table S2; Figs 1 and 3). In *Sorghum* this group included the homologs of the genes PsACR|H.14, PsACR|H.13, PsACR|H.12, PsACR|H.6, PsACR|H.5 and PsACR|H.4.The homologues of the last two exons of SORBI_008G171900 (PsACR|H.14b) were re-located immediately downstream of the first exon of the gene SORBI_008G171400 (PsACR|H.14a) indicating the occurrence of a translocation within the ACR (Supplementary Table S2; Fig. 3). In *Setaria* the homology group was delineated by the homologs of the genes PsACR|H.5, PsACR|H.6, PsACR|H.12, PsACR|H.13 and PsACR|H.14. The translocation that generated the hybrid gene PsACR|H.14 of *Paspalum* was also detected for the related homologs of *Setaria* (Fig. 3) as well as rice, maize and *Brachypodium* (Supplementary Table S2). Similarly to what is reported for the contig PS127F6_c1, the homology regions of genes contained in the contig PS366H1_c1, are mainly confined to the ORFs and in small intronic areas in *Setaria* and *Sorghum* (Fig. 3). The large-scale inversion that affected *Setaria* and *Paspalum* on one side and *Sorghum*, on the other, was also evident for the latter contig (Fig. 3). Of the 3 predicted genes contained in the contig PS366H1_c5, only one showed relevant homology with related genes of *Brachypodium*, maize and rice and none of them showed matching sequences in TAIR database. Scarce homology detected in this contig could be related to sequence divergence that generated areas of hemizygosity on which apomixis-specific SCARs could be developed. The homologs of the two genes (H10_950 and H10_534), belonging to the apomixis-linked BAC H10 previously sequenced^17^, are also located in the same chromosome areas related to apomixis of each of the 5 reference genomes even though they are positioned between 2.2 (*Sorghum*) and 7.8 (maize) Mb respectively away from the conserved main homology areas (Supplementary Table S2). Based on the distance of the homologs at the extremities of the ACR-related area in *Sorghum* and *Setaria*, we estimate the total length of the ACR be around 2.2 Mb and 1.6 Mb respectively. Then, the contigs sequenced and analysed in this study (0.16 Mb) correspond approximately to between 7% and 10% of the total length of the apo locus. To sum up, the two apomixis-linked BACs described here (366H1 and 127F6), together with the previously reported H10, show strong synteny with a chromosome area in the telomeric positions of chromosomes 12, 8, 3, and 4 of rice, *Sorghum*, *Setaria* and *Brachypodium*, respectively, together with a more centromeric position of chromosome 1 in maize. Both large- and small-scale rearrangements affected the ACR of *Paspalum* compared to the related areas of the reference genomes considered. While the large-scale inversions detected likely derived from grass speciation (see GRAMENE database at http://www.gramene.org/), the small-scale translocations, such as those affecting genes PsACR|F.7 and PsACR|H.14, are likely *Paspalum*-specific and could have arisen either at the *Paspalum* divergence or because of polyploidization.

### *In situ* hybridisation analysis

**Figure 4:**
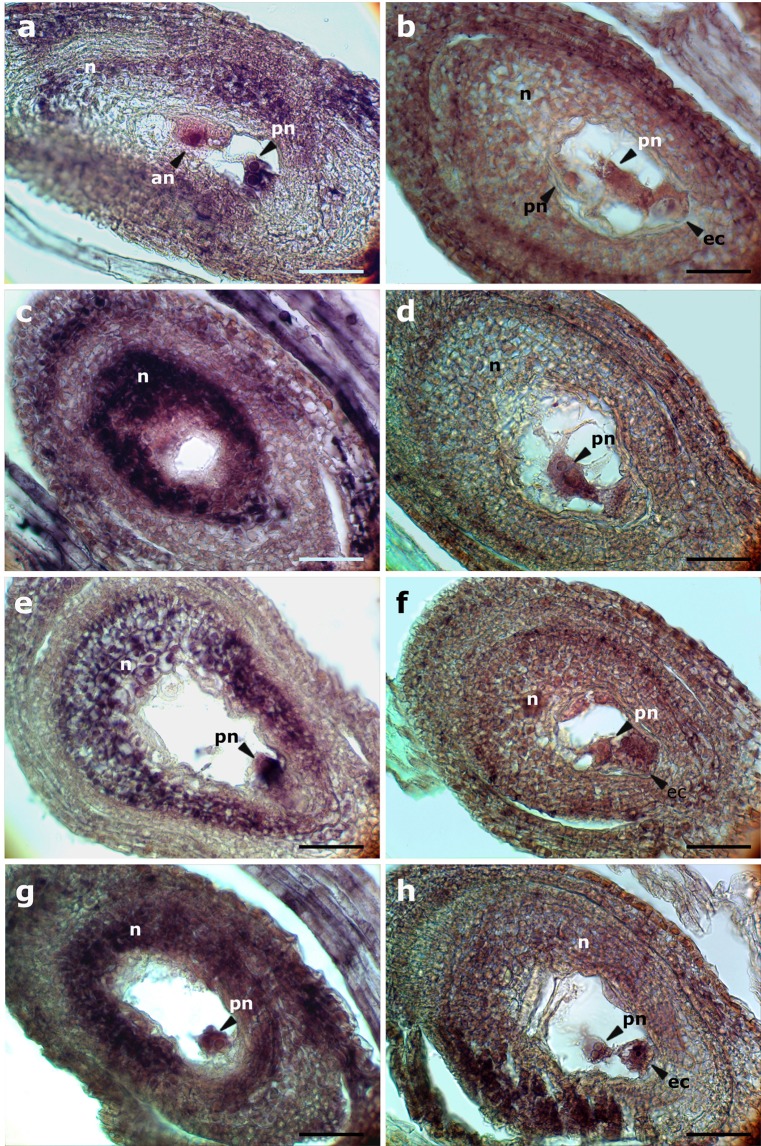
*In situ* hybridisation analysis of PsACR|H5 (**a**–**d**) and PsACR|H.13 (**e**–**h**) transcripts in female reproductive tissues at anthesis of sexual and apomictic *P*. *simplex*. Sections of sexual (**a**,**e**) and apomictic (**b**,**f**) ovules hybridised with antisense probes (detecting the sense transcripts); sexual (**c**,**g**) and apomictic (**d**,**h**) ovules hybridised with the sense probes. an = antipodal cells, ec = egg cell, n = nucellus and pn = polar nuclei. Bar = 50 μm.

Based on *in silico* mapping, we noticed that some of the genes contained in the apomixis-linked BACs of *P*. *simplex* belong to a kind of “ancestral” chromosome segment homologous to the subtelomeric region of rice chromosome 12 (genes PsACR|F.7, PsACR|F.15, PsACR|H.12, PsACR|H.13, PsACR|H.14 and both genes of BAC H10), while others (for example genes PsACR|F.14, PsACR|H.5, and PsACR|H.6) migrated from different parts of the grass genome and contributed to form a more recent syntenic group homologous to the telomeres of the newly formed chromosomes 8 and 3 of *Sorghum* and *Setaria*, respectively. Furthermore, as both sense and antisense transcripts were detected in the female reproductive cell lineages for the pseudogene PsACR|H.6 (*PsORC3*^19^), we wondered whether antisense-mediated regulation of gene expression could be related to the condition of pseudogene. To address this hypothesis, *in situ* hybridisation of the following genes located on the portion of the ACR represented by the contig PS366H1_c1 was undertaken on apomictic and sexual flowers of *P*. *simplex* at anthesis: *i*) gene PsACR|H.5 (encoding for a F-box domain containing protein) as representative of newly migrating functional genes and, *ii*), gene PsACR|H.13 (similar to a PPR repeat containing gene) as an “ancestor” non-functional gene (Supplementary Table S2). The antisense probe for gene PsACR|H.5 (detecting the sense transcript), revealed a signal in polar nuclei and antipodals in sexual (Fig. 4a) and polar nuclei in apomictic ovules (Fig. 4b). The sense probe (detecting the antisense transcript) showed an intense hybridisation signal in the nucellus (Fig. 4c), and polar nuclei (Supplementary Fig. S2a) of sexual ovules. Conversely, the same probe showed no signal in nucellus and a strong one in polar nuclei of apomictic ovules (Fig. 4d). The gene PsACR|H.13 was expressed as sense transcripts in the nucellus, polar nuclei (Fig. 4e), egg cell (Supplementary Fig. S2b) and antipodals (Supplementary Fig. S2c) of sexual ovules, and in polar nuclei, egg cell (Fig. 4f) and antipodals (Supplementary Fig. S2d) of apomictic ovules. A strong hybridising signal related to antisense transcripts was detected in the nucellus, polar nuclei (Fig. 4g), and antipodals (Supplementary Fig. S2e) of sexual ovules, and in polar nuclei, egg cell (Fig. 4h),and antipodals of the apomictic ones (Supplementary Fig. S2f). Although multiple aposporic embryo sacs are a distinctive character of apomictic reproduction in *P*. *simplex*; these are normally detected at early stages of development. Usually at the stage of anthesis, fewer or more often a single embryo sac is contained in mature apomictic ovules^30^.

In conclusion, both genes are expressed mainly in nucellus and polar nuclei indicating that they are probably subjected to an extent of coordinate expression. Furthermore, as these genes expressed both sense and antisense transcripts in apomictic and sexual phenotypes, no relationships could be evidenced between gene migration and expression mode for the same genes.

### Real Time RT-PCR

**Figure 5:**
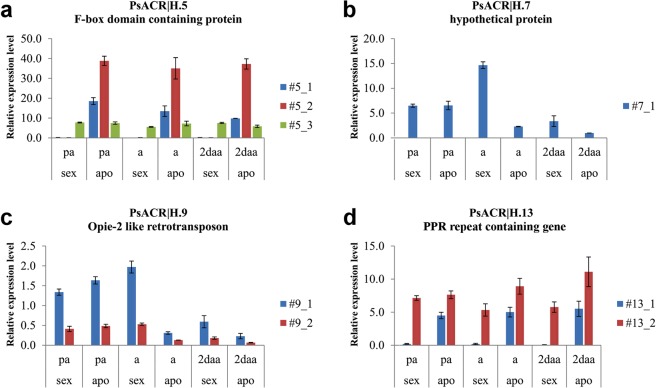
Determination of the transcriptional profile of 4 apomixis-linked genes through qRT–PCR assay carried out at three development stages. pa = pre-anthesis, a = anthesis, 2daa = 2 days after anthesis, sex = sexual plant, apo = apomictic plant. Error bars indicate the SEs.

To investigate whether quantitative differences on gene expression between apomictic and sexual flowers could be related to the condition of pseudogene, Real-Time qPCR assays were performed on 4 genes of the contig PS36601_c1, including PsACR|H.5 and PsACR|H.13 used for *in situ* analyses and the two additional genes PsACR|H.7 and PsACR|H.9. The two genes PsACR|H.5 and PsACR|H.7 are predicted to encode for functional proteins, whereas PsACR|H.9 and PsACR|H.13, likely expressed non-coding transcripts. On the basis of the presence of phenotype-specific SNPs on the cloned alleles (see M&M), we amplified 2 apomixis-specific alleles for the gene PsACR|H.5 (#5_1 and #5_2; Fig. 5a) and one apomixis-specific splicing variant for PsACR|H.13 (#13_1; Fig. 5d). No sex-specific alleles could be amplified, as sexual genomes are shared between apomictic and sexual genotypes of *P*. *simplex*. As a consequence of this, a single allele common to both genotypes was detected for the genes PsACR|H.5, PsACR|H.7 and PsACR|H.13 (#5_3, #7_1 and #13_2 respectively; Fig. 5a,b,d) and 2 for the gene PsACR|H.9 (#9_1, #9_2; Fig. 5c). Specificity was confirmed by partial non-overlapping expression patterns. More in detail, for the gene PsACR|H.5, while the allele variant # H.5_3 displayed uniform expression in all investigated samples, #H.5_1 and #H.5_2, were highly expressed in apomictic florets and showed no expression in the sexual ones (Fig. 5a). The expression of the single allele #H.7_1 was identical in both reproductive phenotypes at pre-anthesis, increased at anthesis in sexual florets and decreased in post-anthesis (Fig. 5b). Regarding the gene PsACR|H.9, while no difference in transcript abundance was detected between sexual and apomictic flowers at pre-anthesis, both alleles were dramatically down regulated in apomictic florets at anthesis and in both phenotypes at post-anthesis stages (Fig. 5c). For the gene PsACR|H.13, our investigations allowed to identify a splicing variant that forms a transcript with a longer coding sequence compared to the expected one. The expression of this splicing variant (#13_1; Fig. 5d) was clearly detectable in all apomictic samples, with little or no expression variation among developmental stages, whereas it was undetectable in all sexual samples. The relative abundance of the common allele # 13_2 was similar in sexual and apomictic samples in pre-anthesis stages, while it was up regulated in apomictic flowers at both anthesis and post anthesis stages (Fig. 5d).

To sum up, apomixis-specific allelic variants were characterized by constitutive expression across developmental stages considered. Furthermore, no obvious relationship was found between expression pattern of the selected apomixis-linked genes and their degeneration.

### *In silico* gene expression analysis

To investigate whether the apomixis-linked genes of *P*. *simplex* analyzed here are expressed in reproductive tissues and validate the expression pattern of those analysed by RT-qPCR, their sequences were queried onto the available 454/Roche reference flower transcriptome database of the related species *P*. *notatum*^31^. In doing so, expressed sequences were found for 20 out of the 34 apomixis-linked genes present in the BACs (Supplementary Table S3). A total of 34 isogroups were detected. Most isogroups (82.35%) showed 1–4 transcript variants (isotigs) that could represent specific allelic forms or splicing variants expressed during the reproductive development. Six genes (17.65%) showed 5 or more (up to 19) isotigs. Of the 36 isotigs, 19 corresponded to *Arabidopsis* orthologs. The detection of multiple isotigs for the same gene could be associated with the heterozygous and polyploid nature of both *P*. *simplex* and *P*. *notatum*.

Out of the 36 isotigs detected, six showed differential representation between the apomictic and sexual databases (Table 2): two of them (isotigs 9721 and 13832, homologous to genes PsACR|H.7 and PsACR|F.3, respectively) were less represented in the apomictic database and four (isotigs 9764, 20690, 10887 and 27671, homologous to genes PsACR|H.5, PsACR|H.13, PsACR|F.14, and H10_950, respectively) were less represented in the sexual database (Table 2). Isotigs13832 and 9764 are significantly overexpressed in sexual and apomictic flowers, respectively (logFC > |2| and FDR < 0.05). Both transcripts deserve further analysis to prove their association with the corresponding phenotype. The case of gene PsACR|H.13 deserves a separate description. Three isotigs, namely 20690, 28985 and 43473 were identified for this gene (Supplementary Table S3). Of these, isotig 20690 is up regulated in sexual sample compared to the apomictic one (Table 2), whereas the other two did not differ for their expression between the two phenotypes. This expression pattern mirrored that of the RT-qPCR of *P*. *simplex* according to which there is an allele specifically expressed in apomictics (PsACR|H.13_1, Fig. 5d) and the other expressed in both phenotypes. This scenario is consistent with the presence in both species of apomixis-specific alleles together with others that are shared between the sexual and apomictic phenotypes.

**Table 2:** Expression analysis of the apomixis-linked genes of *P*. *simplex* and their *P*. *notatum* homologs.

***P***. ***notatum*** **Roche-454 transcriptome expression analysis**						
Isotig ID	13832*	20690	27671	10887	9764*	9721
Reads Apo^a^	0.987 (1)	9.873 (10)	86.856 (88)	8.883 (9)	12.831 (13)	67.116 (68)
Reads Sex^a^	17.204 (17)	2.024 (2)	49.588 (49)	1.012 (1)	0	148.764 (147)
logFC	3.894	−2.287	−0.879	−3.054	−6.732	1.075
logConc	3.974	3.522	6.663	3.329	3.611	3.611
p-value	1.46e^−04^	3.86e^−02^	6.06e^−04^	2.15e^−02^	2.45e^−04^	2.57e^−07^
FDR	5.57e^−03^	3.83e^−01^	1.80e^−02^	2.70e^−01^	8.49e^−03^	2.07e^−05^
***P***. ***simplex*** (**in RT-qPCR assays**)						
Gene ID	PsACR|F.3	PsACR|H.13	H10_950	PsACR|F.14	PsACR|H.5	PsACR|H.7
RUE^b^ Apo	nd	5.01	nd	nd	37.04	3.27
RUE^b^ Sex	nd	0.16	nd	nd	0.13	8.18
p-value	nd	3.68e^−05^	nd	nd	1.81e^−06^	2.76e^−02^
FDR	nd	7.36e^−05^	nd	nd	7.25e^−06^	3.68e^−02^

^a^Normalized number of reads in apomictic (A) and sexual (Sex) 454/Roche libraries. In brackets are the non-normalized read count values; *Transcripts showing significant differences in abundance between apomictic and sexual libraries (logFC > |2| and FDR < 0.05); ^b^Relative Units of Expression.

Most of the genes contained in the apomixis-linked BACs of *P*. *simplex* are expressed in flowers of *P*. *notatum* regardless of their nature of pseudogene. Considering the limited number of genes analysed, there is a good agreement between the expression pattern of the apomixis-linked genes of *P*. *simplex* and their homologs of *P*. *notatum*.

## Discussion

As grass genomes are largely collinear and apomixis is spread across the family, several authors have hypothesized it might be controlled by the same set of genes wherever it occurs^32^. However, while the genomic regions controlling apomixis appear to be similar within genera^15,33^, the apomixis-controlling loci seem to be highly divergent between them^34^. Such interpretative framework is consistent with the observation that apomixis has originated many times independently in the grass family^35^, whereas it spreads among the several species of the same genus by intra or inter-specific hybridisation^36^. In any cases, these findings render the identification of the genetic determinants of apomixis difficult. In *P*. *simplex*, comparative sequence analysis of genes contained in apomixis-linked BACs clearly points to a genomic region that is syntenic among the five main reference grass species^3,23^ and is located in a telomeric position on chromosome 12, 8, 3 and 4 of rice, *Sorghum*, *Setaria* and *Brachypodium*, respectively, and in centromeric area of maize chromosome 1. In particular, the *Setaria* chromosome 3, together with chromosome 7, originated through a series of translocations and inversions involving ancestral chromosomes similarly to what concerned the actual chromosomes 4, 5, 12 of rice and 6, 8, 9 of *Sorghum*^23^. An 840 Kb inversion was reported in this genomic area of *Setaria*, with respect to its orthologous regions of rice and *Sorghum*^37^ and, similarly, a large-scale inversion was documented in the same region of *Brachypodium* (http://www.gramene.org/). Although the limited portion of the ACR analysed does not allow a generalization, we argue that the origin and structure of the apomixis locus of *Paspalum* shares several commonalities with other multi-gene complexes, such as that related to the Y-chromosome of dioecious plants. Above all, among them are repression of recombination, presence of TE and gene degeneration^38^. The Y-chromosome originated from autosomal chromosomes by initial suppression of recombination in the regions containing the sex controlling genes. Further expansion and rearrangement of the non-recombining Y-locus together with migration of male determining genes caused chromosome heteromorphism and dioecism^39^. Such expansion of recombinational suppression occurred stepwise across chromosomes producing a kind of stratification of the strength of recombination suppression^40^. Such structural stratification is recognizable in the chromosomes 12 of rice and 8 of *Sorghum* harbouring the syntenic group to the apomixis locus of *Paspalum*^41^. From a functional point of view, the evolution of Y-chromosome induces both the silencing of the female genes through the action of degenerated genes and, at the same time, the development of male-specific organs and function by the action of master functional genes^39^. In this sense, the female phenotype is recognized as the default state in some dioecious systems^42^, as well as the sexual phenotype is considered the default state in apomictic systems^43^. In a more general view, the ACR of *Paspalum* shares commonalities with operon-like gene clusters that in plants control complex traits such as those designated to the production of secondary metabolites mainly involved in plant defense^44^. Gene clusters are commonly defined as a set of two or more non homologous functionally related genes that share a close genomic neighbourhood^45^. Genes contained in a cluster can be transcribed independently or subjected to various extents of co-expression^45^. If clustered genes are organized as a single transcriptional unit they are defined as operon^46^. While operons are specific of prokaryotic genomes and likely originated by horizontal gene transfer^47^, genes clusters are reported for many eukaryotes including plants^48^ and evolved *de novo* by initial gene duplication followed by neo- or sub-functionalization and genome rearrangements of various nature^49^. Among the several features common to gene clusters is their origin from subtelomeric dynamic regions characterized by high rates of gene rearrangements^44^. Furthermore, a segmental duplication followed by gene inversion and recruitment has been proposed as origin of gene clusters required for the synthesis of triterpenes in Arabidopsis^50^. As a consequence of these rearrangements, the genes within these clusters are coordinately expressed at the chromatin level and associated with repressive marks^44^.

Another point of similarity between the ACR of *Paspalum* and multi gene complexes is related to gene expression. Two of the apomixis-linked genes considered here (genes PsACR|H.5 and PsACR|H.13), together with the previously analysed *PsORC3*^19^, are expressed as sense and antisense transcripts in reproductively committed cell lineage indicating they might be regulated by the same promoter. Furthermore, at least for the apomixis-linked *PsORC3* allele, its repressive role on the expression of its sexual counterpart is evidenced^19^. Finally, recent studies showed that the parthenogenetic development of the embryo in *Paspalum* is superimposed over the sexual one by a mechanism mediated by DNA methylation indicating that the ACR might be subjected to a chromatin-mediated gene silencing mechanism^51^.

Conversely, TE accumulation is not a common feature between apomixis loci and multi gene complexes. First sequencing efforts of apomixis loci led to the observation that there was an unusual accumulation of TEs in these loci^52^_._ This fact suggested that TEs may act as a sink to sequester factors involved in sexual reproductive pathway and possibly causing apomixis^53^. Nowadays, as WGS of the most representatives of the grass clades become available, we can conclude that in *Paspalum* as well as in other natural apomictic systems^54^, the proportion of repeated elements between apomixis-linked BACs and that detected in the whole genome were not different^24^. Furthermore, an extensive repetitive structure associated with apomixis was confirmed to be dispensable to express the apomictic phenotype in *Hieracium*^55^.

To sum up, as in several multi gene complexes, the ACR of *Paspalum* originated from chromosomically unstable subtelomeric regions that experienced both large and small scale inversions. Hence, the rise of apomixis in *Paspalum* could be the consequence of: (*i*) a casual grouping of a series of sexual genes in the same genomic context during speciation and (*ii*) a polyploidization event that generated a divergent and recombinationally blocked chromosome segment that harboured both pseudogenes, which in some cases silenced their sexual counterparts, and functional genes that evolved the specific functions of apomixis development.

However, apomictic reproduction cannot be considered only as a short-circuited sexuality, especially in the case of aposporic apomixis, where the development of aposporic embryo sacs does not necessarily imply the suppression of meiosis that parallels the development of aposporic initials until the formation of megaspores. Thus, genetic determinants for “gain of function” should be present in the apomixis locus of *Paspalum* as it was observed in other apomictic systems for the parthenogenesis^56^. Finally, a gain of function could derive from negative regulation of silencer genes such as those required for the repression of the development of additional embryo sacs from nucellar cells^57^ and/or the autonomous endosperm development in the absence of fertilization^58^.

Our findings contribute novel information on the genetic structure and nature of the ACR in *Paspalum* which, due to its clear affinity with corresponding syntenic groups of grass model systems make this genus a unique model to study apomixis. Furthermore, in the perspective to introgress apomictic reproduction in sexual crops, even if the master genes of apomixis might be few, the sexual recipient genome should be prepared to regulate a *plethora* of genes acting downstream of the apomixis linked factors. Therefore, when attempting to develop an apomictic system in sexuals it is necessary to use the closest natural apomictic relative as source of master genes. In this perspective, and on the basis of the results reported here both *Setaria* and *Sorghum* are more suitable then rice as target crops to develop an artificial apomixis system based on genes isolated from *Paspalum*. In any cases, this genus is an excellent biological system *per se* as it includes both sexual and apomictic cytotypes of important forage crops.

## Materials and Methods

### BAC isolation and sequencing

The two apomixis-linked BAC clones, 366H1 and 127F6 were initially isolated from a genomic BAC library of apomictic *P*. *simplex* using SCARs co-segregating with apomixis^28^. Thirty-nine of the 41 BACs positive to apomixis-linked SCARs were isolated using a plasmid Purification Maxi Kit with the low-copy plasmid/cosmid protocol (Quiagen) and blotted onto Hybond-N^+^ membranes using a Bio Dot Microfiltration Apparatus (Bio Rad) according to the supplier’s instructions. The blots containing 200 ng of each plasmid were hybridized to radio-labelled ACR-specific probes as reported^15^. The selected BACs were sequenced using the 454 pyrosequencing system (GS FLX, 454 Life Sciences) reaching a six fold coverage. The final sequence was assembled using Sequencher v4.0 software (Gene Codes Corporation, http://www.genecodes.com) set to an overlap minimum of 20 bp with 95% identity and annotated based on the software packages Fgenesh + with a monocot Markov model and GeneID^59^. The accuracy of the assembly was validated by comparing the predicted and actual restriction digestion profiles for a subset of restriction enzymes. Consensus gene models were derived by comparing the gene models with a reference protein database (UNIREF90^60^). The annotation of all identified homologous genes was performed with the CLC Genomics Workbench v7 (QIAGEN) and by following the guidelines for sequence annotation using GFF files. GFF files were combined with the assembled sequence with ARTEMIS software to generate a graphical output of annotated genes (not shown). The two contigs Ps366H1_c1 and Ps127F06_c1 were searched for repetitive elements together with PsH10^17^ as a comparison and Ps366H1_c5 as this contig contained the hemizygous SCAR marker used to select the corresponding BAC using the repeat masker software version 4.0.6 (http://www.repeatmasker.org/cgi-bin/WEBRepeatMasker) against the Panicoid repeat database using the default settings. PsH10 was obtained by grouping the largest 6 non overlapping contigs so as to reach a size comparable with those of the two larger contigs.

### *In silico* mapping

The GRAMENE database^61^ (http://www.gramene.org) was interrogated with the sequence of each gene with the BLASTx option to retrieve the best hits for each of the genomes of *Setaria italica*, *Sorghum bicolor*, *Zea mays*, *Brachypodium distachyon* and *Oryza sativa* at December 3^rd^ 2018. The genomic sequence of each gene was used to generate data in Supplementary Table S2 and Fig. 1. TAIR database^62^ (https://www.arabidopsis.org/) was interrogated with BLASTx option to retrieve hypothetical gene functions. To generate graphical representation of syntenic relationships, all chromosome regions carrying significant matches with genes included in the ACR were split by using a fixed window of 250 kb and extracted as graphical representations. Circular figures (Fig. 1) were constructed by using J-circos (https://sourceforge.net/projects/jcircos/), following the software guidelines. Since the BAC clones and corresponding syntenic chromosome regions displayed marked length differences, the chromosome sizes needed to set the circos plot backbone were defined by dedicating 45/360 circumference degrees to the *Paspalum* sequences and by leaving the remaining 315/360 circumference degrees to the chromosome regions matched by the BAC clones. Boundaries of genes included in the *Paspalum* sequence clones were plotted using the option: circus_wiggle, as indicated in the software guidelines. Connections between homologous regions were then plotted by using the Circos_bridge function. Line colours were set to discriminate the different chromosomes. Finally, circular figures were exported and manually edited with the GNU Image Manipulation Program (GIMP) v2.8 for proper graphical representation. Figures 2 and 3 were manually edited with PowerPoint to graphically underline syntenic regions and collinearity relationships retrieved from GRAMENE database (BLASTx). In order to identify the BAC’s sequences expressed in reproductive tissue, a BLASTn search was carried out using the *P*. *simplex* gene sequences as query against the annotated floral *P*. *notatum* reference 454/Roche mRNA transcriptome (built from SRX1971037 and SRX1971038 for apomictic and sexual libraries, respectively^31^.

### Expression analyses

Plants used in this study were apomictic and sexual genotypes of tetraploid *P*. *simplex* (2n = 4 × = 40) belonging to a backcross population segregating for apomixis^14^. RT-qPCRs were performed by considering three developmental stages, corresponding to pre-anthesis (2–3 days before anthesis corresponding to stage III^18^), anthesis and two days after anthesis. For RNA extraction, florets were collected separately from a minimum of three genotypes (biological replicates) for each phenotype which were processed individually. Total RNA was extracted from collected samples using the Spectrum™ Plant Total RNA Kit (Sigma-Aldrich) following the protocol provided by the manufacturer. The contamination of genomic DNA was avoided by a DNase I treatment using the On-Column DNase I Digestion Set (Sigma-Aldrich) Kit. The abundance and pureness of RNAs were assessed using a NanoDrop 2000c UV-Vis spectrophotometer (Thermo Scientific, Pittsburgh, PA). The integrity of extracted RNA samples was estimated by electrophoresis on a 0.8% agarose/1 × TAE gel containing 1 × SYBR Safe DNA stain (Life Technologies, Carlsbad, CA, USA). cDNA synthesis was performed starting from 400 ng of total RNA, by using the RevertAid First Strand cDNA Synthesis Kit (Thermo Scientific) following the supplier’s instructions. For each of the 4 selected genes for Realtime PCR, a single primer combination (PsACR|H.gene number_gfor/rev; Supplementary Table S4) was designed on CDS and used for PCR amplification on two apomictic and two sexual DNA samples. The relative amplicons were cloned in pGEM-T Easy vectors and sequenced bidirectionally using SP6/T7 primers. By aligning the resulting sequences with those of the related genes on the BACs, several SNPs were identified. On the basis of these SNPs, primers were designed to amplify each specific allelic variants. The expression of each gene was analysed by using up to three different primer combinations (Hgene number.1–3_for/rev; Supplementary Table S4) designed to assay the expression of multiple alleles and/or splicing variants detected in apomictic and sexual cDNAs. Amplification reactions were performed using StepOne thermal cycler (Applied Biosystems), equipped with 96-well plate systems, and FAST SYBR green Master Mix reagent (Applied Biosystems). Three technical replicates were adopted for each amplification reaction. The amplification efficiency was calculated from raw data using OneStep Analysis software (Life Technologies). Relative amplification performance, expressed as fold change, was calculated with the ΔΔCt method^63^ using cytidine deaminase gene (*PsCDA*; GeneBank accession no. AM400871)^18^ as the internal control (housekeeping). Error bars indicate the standard error observed among the three biological replicates (Fig. 5). Statistical analysis of expression counts in Table 2 was carried out according to the False Discovery Rate method^64^. For *in situ* analysis approximately 20–30 ng of cDNA from flowers of an apomictic genotype were amplified using specific primer pairs for each of the genes PsACR|H.5 and PsACR|H.13 (PsACR|H.gene number_ishfor/ishrev; Supplementary Table S4). The derived 792 bp (PsACR|H.13) and 916 bp (PsACR|H.5) amplicons were cloned into a pGEM-T Easy vector (Promega) and sequenced bidirectionally with SP6 and T7 primers to establish the direction of the insertion. Both probes were labeled using a Roche DIG RNA labeling kit (SP6/T7) and hydrolyzed into 150–200 bp fragments. Spikelets of sexual and apomictic *P*. *simplex* genotypes were collected at anthesis stage, fixed, and embedded in paraffin and used for *in situ* hybridisation, as reported by Siena *et al*.^65^. Detection was performed following the Roche DIG detection kit instructions using anti-DIG AP and NBT/BCIP as substrates. Fourteen sexual and 11 apomictic ovules were observed as hybridised with the antisense probe of the gene PsACR|H.5 and 10 sexual and 4 apomictic ovules with the sense probe of the same gene; 8 sexual and 11 apomictic ovules were observed as hybridized with the antisense probe of PsACR|H.13 and 8 sexual and 11 apomictic ovules with the sense probe of the same gene.

## Supplementary information

Supplementary Figures

Dataset 1

Dataset 2

Dataset 3

Dataset 4

## Data Availability

Supplementary Table S2 provides information on the primers used for the RT-qPCR and *in situ* experiments. The sequence data of the contigs reported in this paper have been deposited in the GeneBank database (https://www.ncbi.nlm.nih.gov/genbank) [accession nos MH106546, MH106547, MH106548, MH106549].
