# Supplementary material for: A Portion of the Apomixis Locus of Paspalum Simplex is Microsyntenic with an Unstable Chromosome Segment Highly Conserved Among Poaceae
Source: Sci Rep. 2019 Mar 1;9:3271. doi: 10.1038/s41598-019-39649-6 (PMC6397161; doi:10.1038/s41598-019-39649-6)

A PORTION OF THE APOMIXIS LOCUS OF PASPALUM SIMPLEX IS MICROSYNTENIC WITH AN UNSTABLE CHROMOSOME SEGMENT HIGHLY CONSERVED AMONG POACEAE

Giulio Galla, Lorena A. Siena, Juan Pablo A. Ortiz, Helmut Baumlein, Gianni Barcaccia, Silvina C. Pessino, Michele Bellucci and Fulvio Pupilli

Figure legends

Supplementary Figure S1. Dot blot of apomixis-linked BACs hybridized with the rice marker c996 as probe.

Supplementary Figure S2. *In situ* hybridization analysis of PsACR|H5 (a) and PsACR|H.13 (b-f) transcripts in female reproductive tissues at anthesis of sexual and apomictic *P. simplex*. Sections of sexual (b and c) and apomictic (d) ovules hybridized with antisense probes (detecting the sense transcripts); sections of sexual (a and e) and apomictic (f) ovules hybridized with the sense probes. an = antipodal cells, ec = egg cell, n = nucellus and pn = polar nuclei. Bar = 50 µm.

Uncropped Supplementary Figure S1. Uncropped blot image of Supplementary Figure S1

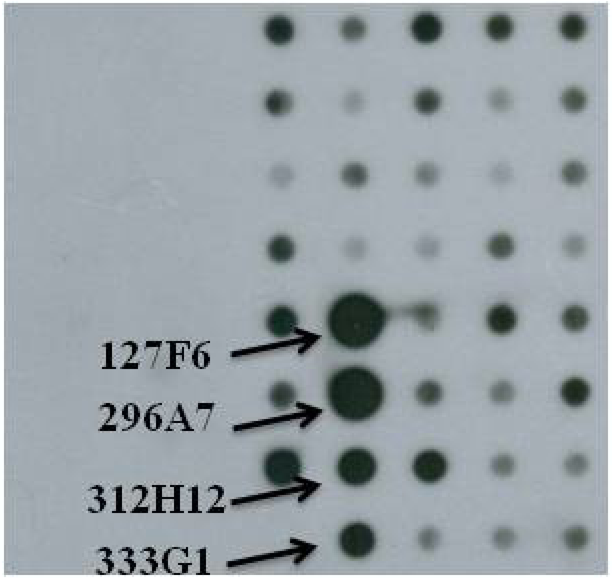
127F6
296A7
312H12
333G1

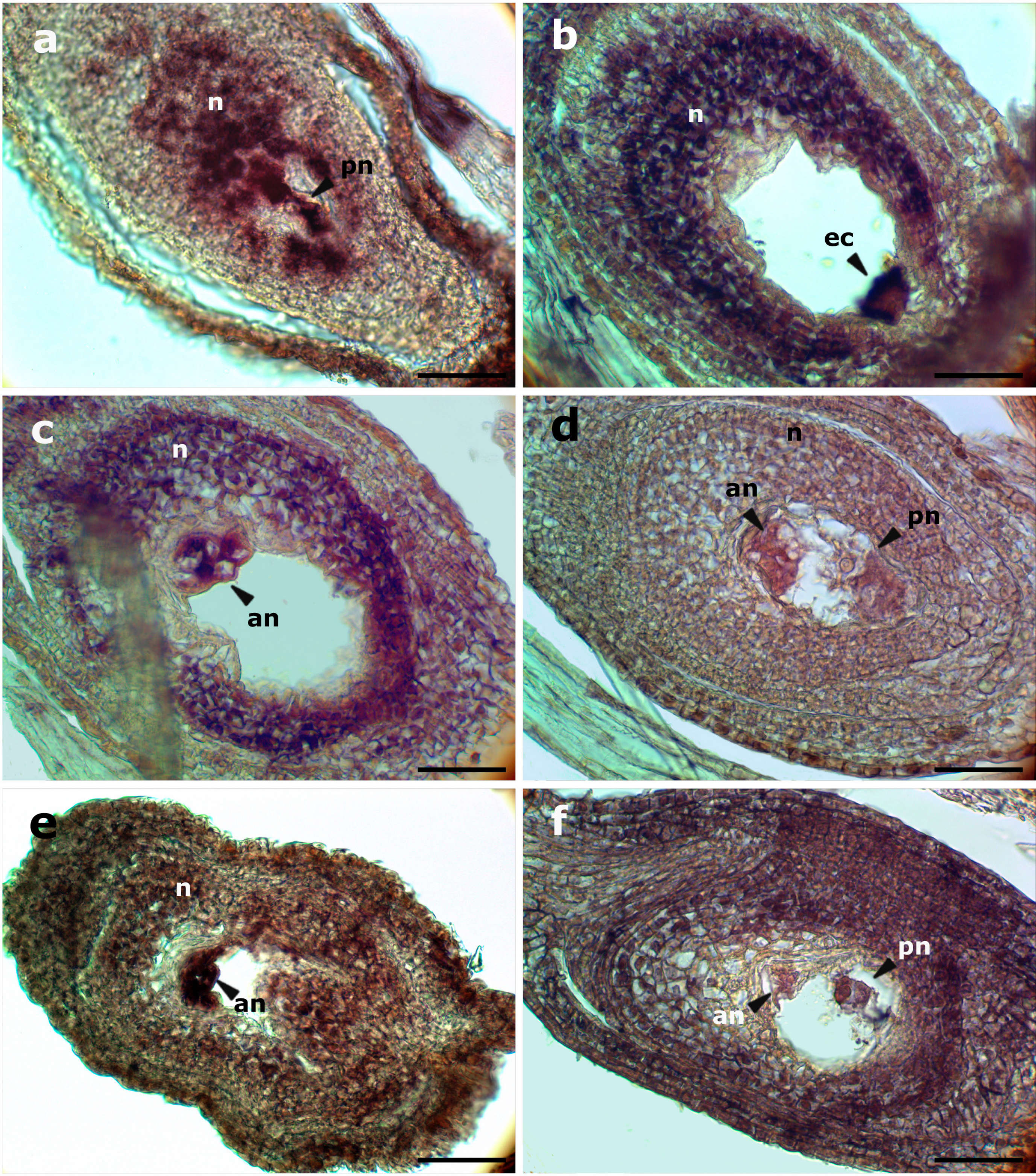
a
n
pn
b
n
ec
c
n
an
d
n
an
pn
e
n
an
f
pn
an

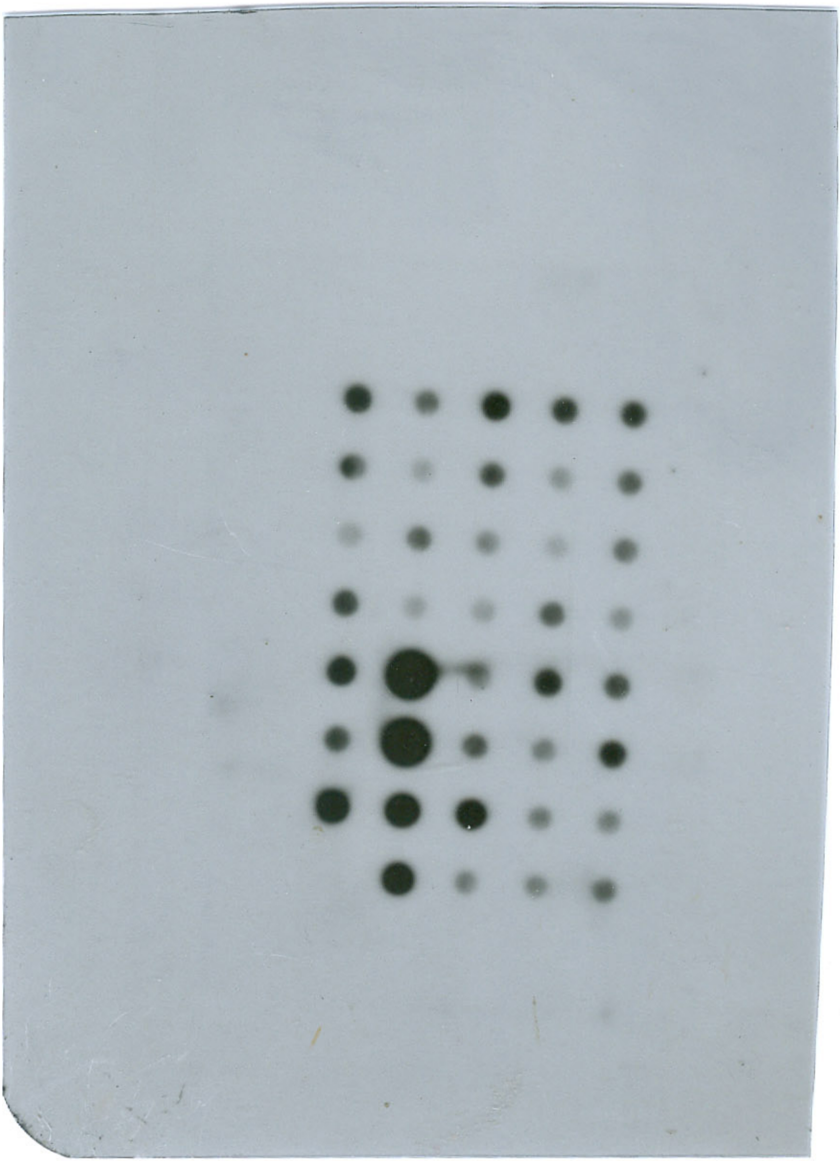

Supplement: Supplementary file 1 — Supplementary Figures [file 41598_2019_39649_MOESM1_ESM.pdf]
